# Detection of neural tube defect in the first and second trimester of pregnancy by ultrasound in Imam Hospital, Ahwaz between December 2008-2010

**Published:** 2012-11

**Authors:** Sara Masihi, Mojgan Barati, Javad Marfu, Zahra Eskandari

**Affiliations:** *Department of Obstetrics and Gynecology, Ahvaz Jundishapur University of Medical Sciences, Ahvaz, Iran.*

**Keywords:** *NTD*, *Intracranial Translucency*, *Anencephale*, *Meningomyelocele*

## Abstract

**Background:** Central nervous system malformations are the second most common congenital malformations after congenital heart diseases. These malformations are associated with many instances of morbidity and mortality which underline the importance of prevention and their early diagnosis.

**Objective:** The objective of this study is the diagnosis of neural tube defect (NTDs) in the first trimester and its comparison to second trimester diagnoses in order to reduce the complications associated with late pregnancy terminations and its costs.

**Materials and Methods:** This study was a trans-sectional study. A total number of 1074 patients who referred to Imam Khomeini Hospital were enrolled in this study. During the 11^th^-13^th^ (+6 days) gestational week the patients were screened sonographically; subsequently they were re-scanned for fetal anomalies during 18^th^-20^th^ gestational week, and we followed the babies after birth. Considering that Intracranial Translucency (IT) was introduced in the newer researches, it was, too, measured in 125 of the cases.

**Results:** In a total number of 1074 patients we had one patient with an anencephalous fetus whom was diagnosed in the first trimester of pregnancy. In the second trimester, we detected one case of myelomenigocele; when we referred to this patient’s first trimester sonography, there was no visible IT. In the 125 cases in whom the IT length was measured, it was normal; the 2^nd^ trimester sonographies in these patients were also normal.

**Conclusion:** It must be noted that the diagnosis of NTD is more accurate in the second trimester of pregnancy. Consequently it is recommended that in high risk patients, the second trimester sonography be performed transvaginally, and in an earlier gestational age (14^th^-16^th^ gestational weeks).

## Introduction

CNS defects are the second most common congenital anomalies after congenital heart diseases. In epidemiologic studies, Neural Tube Defect (NTD) frequency is 1.6 in every 1000 live births. This figure increases to 8 in every 1000 live births in England. These anomalies are associated with many instances of morbidity and mortality, and are therefore important to be diagnosed early in the pregnancy and we realized that the rate of these anomalies were high in our location (Khouzestan). 

In one study in our center the rate of NTDs was 4.2 per 1000 live birth in two years (56 NTDs in 13622 live births). The recurrence rate was 5.4%. The objective of this study is the diagnosis of NTDs in the first trimester and its comparison to second trimester diagnoses in order to reduce the complications associated with late pregnancy termination and its costs. In one study conducted by Lachmann *et al* from 2006-2010, comparing the brainstem diameter and brainstem-occipital bone length between 30 fetuses with open spina bifida (OSB) and 1000 control normal fetuses during 11^th^-13^th^ gestational weeks, it was concluded that most fetuses with OSB had measurable anomalies in their posterior brain. 

In another study conducted by Nicolaides and Chaoui, in England, after using Intracranial Translucency (IT) and Nuchal Translucency (NT) markers, all the studied cases of OSB were associated with Arnold Chiari malformation. By ultarsound, it is possible to diagnose cerebral malformations such as holoporosencephaly, ventriculomegaly, anencephaly, etc. during 11^th^-13^th^ gestational weeks. In a study performed in 2009 by Benoit *et al* using sonographic measurement of IT during 11^th^-13^th^ gestational week to diagnose spina bifida, routine use of midsagittal sonographic view was suggested for early diagnosis of chromosomal defects. Also it was notable that in first trimester sonographies of fetuses with spina bifida, IT was not visible due to the compression of 4^th^ cerebral ventricle by hindbrain.

In another study, 78% of cases who were eventually diagnosed with anencephaly were actually diagnosed as being so, using sonography at the end of the first trimester. Also by ultrasound, spinal defects and cerebral findings associated with meningomyelocele were detectable in 89% of cases who were ultimately diagnosed with meningomyelocele. In a study performed in 2005, the best timeframe for NTD screening was suggested to be the 2^nd^ trimester (92.1%). The percentage goes down to 83% if the screening is performed during the first trimester. It is also recommended to use serum markers such as MSAFP (Maternal Serum Alpha Fetoprotein) which leads to a diagnosis rate of 98%.

In another study in Netherlands 2001, the best time for screening Down’s syndrome and/or NTDs was between 18^th^-21^st^ gestational weeks, using routine prenatal sonography. This method has yielded 94% diagnosis rate; if used in the first trimester, the rate of NTD diagnosis was 76 percent of the cases with a final diagnosis of NTD. In 60-70% of fetuses with 21 chromosome trisomy, the nasal bone is not detectable in sonography during 11^th^-13^th^ (+6 days) gestational week.

## Materials and methods

This descriptive trans-sectional study was performed from December 2008-2010 in Imam Hospital in Ahvaz. 1074 patient was entered the study. We performed NT scan at 11-13W+6 days with specific attention to anatomy of fetus. We repeated the scan at 18-22 weeks. We followed the patients till delivery of fetus. Considering that IT was introduced in the newer researches, it was, too, measured in 125 of the cases and we did not measured it in all of the cases. 

A considerable number of patients who referred to the clinic with a sonographic report of NTD were excluded because they were not visited in their first trimester of pregnancy. The sonography machine models used were Mylab60 and Medison x_10_, and the sonography procedure was performed by two gynecologist with FMF license; if considered necessary, the patients were referred to a fellow sonographist to be rechecked (left picture NT. right picture IT in one of our patients).

## Results

Total number of individuals studied was 1074 cases, 4% of pregnancies were multiple-pregnancies. The average age of the pregnant women was 28.6 years. 2.3% of the cases had NT lengths over 95% percentile (based on FMF software). 

In our cases we had one anancephalic fetus that was diagnosed in the first trimester; this patient was a 40 year old Woman who had history of another anencephalous in first gestation, and had developed gestational diabetes in her recent pregnancy. In the second trimester, we detected one case of myelomenigocele in a 26 year old primigravida woman. 

**Figure 1:**
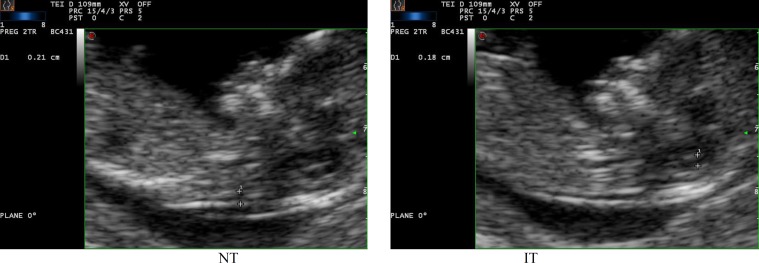
Nuchal Translucency (NT) and Intracranial Translucency (IT) length was measured by ultrasound

**Figure 2:**
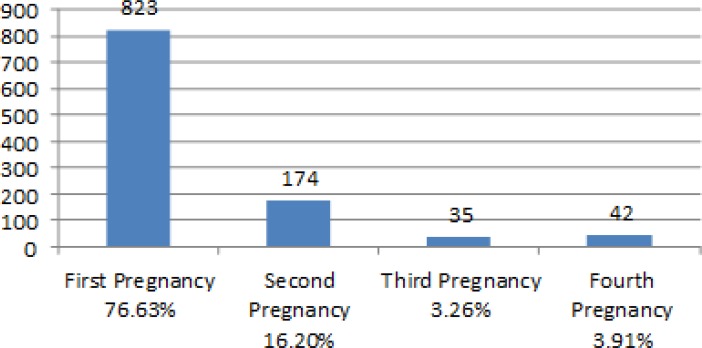
Patient frequency based on total number of pregnancies (gravida).

**Figure 3:**
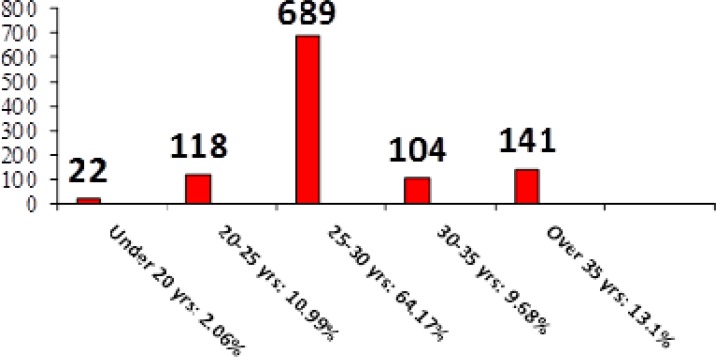
Patient frequency based on age group

**Figure 4:**
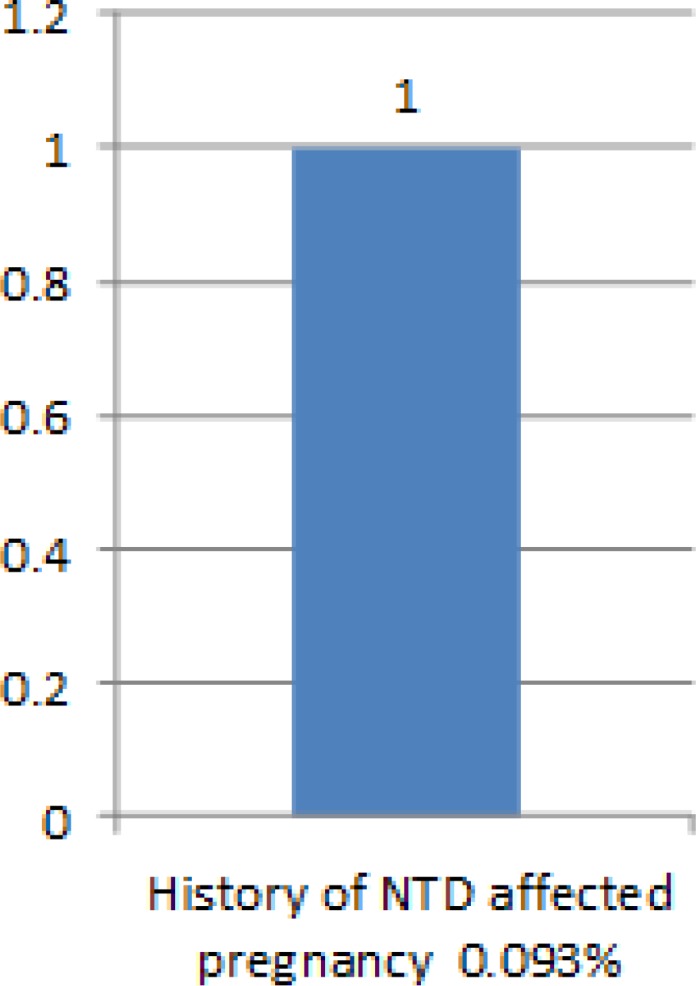
Patient frequency based on history of NTD in previous pregnancies:

**Figure 5:**
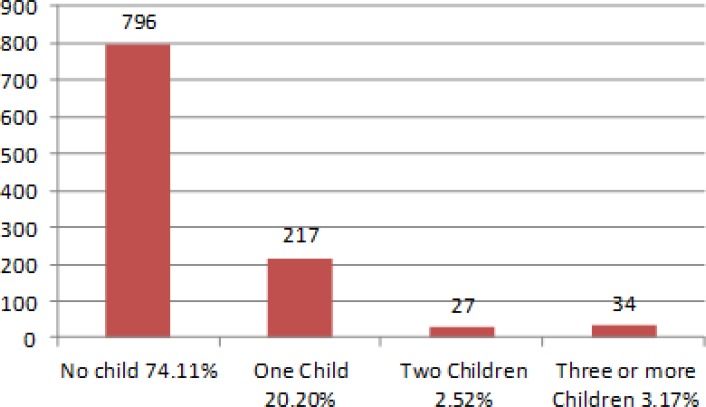
Patient frequency based on number of children

**Figure 6:**
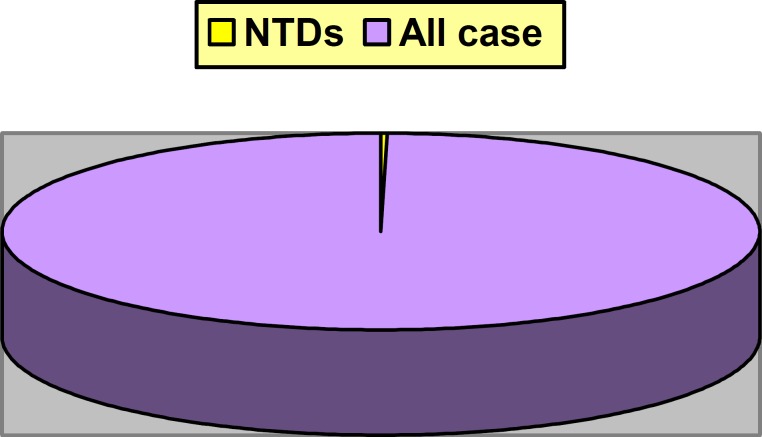
Affected cases

Albeit at the time, we weren’t measuring IT length; subsequently, when we referred to her first trimester sonography, there was no visible IT. In the 125 cases in whom the IT length was normal, the 2^nd^ trimester sonographies were also normal; some of them hadn’t yet delivered their newborns (Figures 1-6).

(There was only one case with a history of NTD in our study: a 40 year G4 patient with a history of anencephaly in her first pregnancy).

## Discussion

Numbers of cases with NTDs were so small that we could not do any statically analysis. Many affected cases that referred to us were in second or third trimester and we had not evaluated them in the first trimester and inevitably they have been excluded. In our 1073 cases we diagnosed one enencephaly and terminated the pregnancy in first trimester by using vaginal prostaglandin in a mother with past history of 3 times C.S. In the end of study we were beginning the measurement of IT in first trimester and unfortunately only in 125 cases this measurement were done. In all of them it were visible and second trimester sonograghy were normal. Prospective large studies will determine the proportion of affected fetuses presenting with absent IT and the extent to which the 11-13 week scan can provide an effective method for early diagnosis of open spina bifida.

In the 1970s, the main method of screening for trisomy 21 was by maternal age and in the 1980s it was by maternal serum biochemistry and detailed ultrasonographic examination in the second trimester. In the 1990s the emphasis shifted to the first trimester when it was realized that the great majority of trisomic fetuses have increased NT that can be detected easily in a mid-sagittal plane of the fetal face at 11-13 weeks. Improved performance of screening was achieved subsequently with the observation that in the same mid-sagittal plane as for measurement of NT it was possible to examine the nasal bone, which is often absent in trisomic fetuses. It is now clear that in this same mid-sagittal plane the fourth cerebral ventricle is easily visible as an IT and that at least in some cases of open spina bifida, caudal displacement of the brain is evident from the first trimester, resulting in loss of the normal IT.

## Conclusion

With the screening methods available, the diagnosis of NTD in the first trimester of pregnancy can lead to a decrease in maternal complications associated with pregnancy termination and its costs. Considering newly proposed markers such as IT and posterior brain measurements, we recommend measuring these markers during the first trimester of pregnancy. Also, because NTD diagnosis is more accurate during the 2^nd^ trimester of pregnancy, it is preferable that the 2^nd^ trimester sonography in high risk patients be performed in earlier weeks of the trimester (14^th^-16^th^) and preferably transvaginally.
